# A case of eosinophilic pustular folliculitis involving palmoplantar successfully treated by apremilast

**DOI:** 10.1093/skinhd/vzaf019

**Published:** 2025-04-07

**Authors:** Jing Xu, Yue-meng Wu, Shang-shang Wang

**Affiliations:** Department of Dermatology, Huashan Hospital, Fudan University, Shanghai, China; Department of Dermatology, Huashan Hospital, Fudan University, Shanghai, China; Department of Dermatology, Huashan Hospital, Fudan University, Shanghai, China

## Abstract

Eosinophilic pustular folliculitis (EPF) is a rare inflammatory dermatosis that predominantly affects seborrheic areas. The condition’s pathogenesis is linked to T helper 2-driven eosinophilic inflammation. We presented a man with EPF involving the face, palms and soles, successfully treated by apremilast, a phosphodiesterase-4 inhibitor. Apremilast’s efficacy in this case adds to emerging evidence supporting its use in EPF, particularly when traditional treatments fail.

## Teaser

We presented a man with EPF involving the face, palms and soles, successfully treated by apremilast, a phosphodiesterase-4 (PDE4) inhibitor. Apremilast’s efficacy in this case adds to emerging evidence supporting its use in EPF, particularly when traditional treatments fail.

**What is already known about this topic?**

- Eosinophilic pustular folliculitis (EPF) is a rare inflammatory dermatosis that predominantly affects seborrheic areas. There is still a lack of standardized therapeutic protocols.

**What does this study add?**

- Apremilast emerged as a potential treatment option to EPF due to its anti-inflammatory effects.

## Introduction

Eosinophilic pustular folliculitis (EPF) was first described in 1965 by Ofuji and is characterized by recurrent papulopustular eruptions, typically involving seborrheic areas of the body such as the scalp, face and trunk.^1^ It is a rare inflammatory condition, with several subtypes, including classical EPF, immunosuppression-associated EPF and infantile EPF.^2^ Although lesions are primarily confined to hair-bearing areas, atypical presentations involving non-seborrheic sites such as the palms, soles and nails are exceedingly rare.^3^

The exact pathogenesis of EPF remains incompletely understood, but it may be a non-specific response to various antigenic stimuli that impair the immune system.^4^ This inflammatory milieu causes pustule formation, tissue damage and the chronic relapsing nature of the disease. Given the rarity of the condition, especially when involving non-seborrheic areas, EPF is often misdiagnosed as other dermatoses like palmoplantar pustulosis or psoriasis, leading to delays in appropriate treatment.

Management of EPF remains challenging due to its relapsing nature and the lack of standardized therapeutic protocols. Treatments ranging from indomethacin and dapsone to corticosteroids and immunomodulators have been utilized with varying success.^5^ Recently, apremilast, a selective inhibitor of phosphodiesterase-4 (PDE4), has emerged as a potential treatment option due to its anti-inflammatory effects. This case report describes a patient with EPF involving the face, palms and soles who responded favourably to apremilast after not improving with conventional treatments.

## Case report

**Figure 1:**
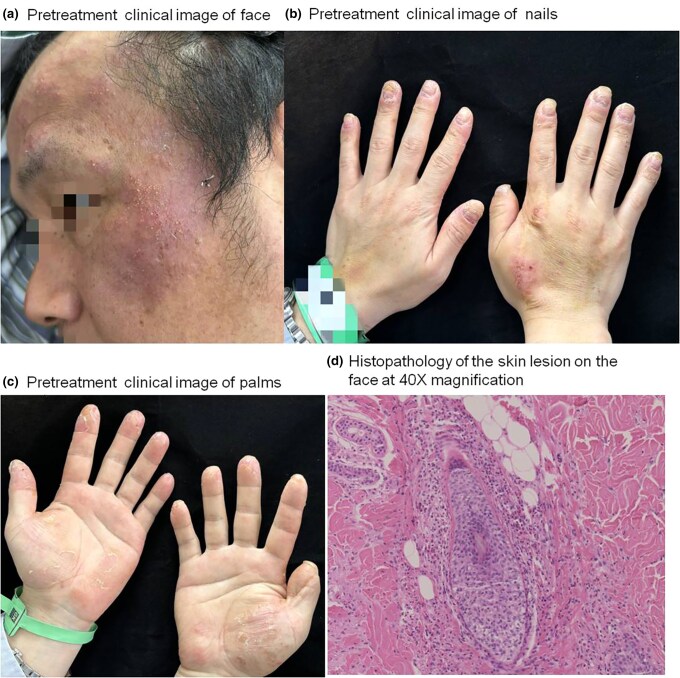
Pretreatment clinical presentation of the eosinophilic pustular folliculitis (EPF) patient: (a) face, (b) nails and (c) palms. (d) Pathological appearance of biopsy specimens from the face.

A 40-year-old man presented with a 6-month history of pruritic erythematous papules and pustules on the palms and soles, with subsequent involvement of the face 1 month later. The patient reported exacerbation of the lesions after wearing rubber gloves, although no definitive triggers were identified. In addition to cutaneous manifestations, the patient also exhibited periungual erythema and nail deformities, including thickening and ridging (pachyonychia) (Figure 1a,b,c).

Initial treatment with acitretin (an oral retinoid), topical triamcinolone acetonide, and econazole nitrate cream and tacrolimus ointment for more than 6 weeks provided no significant improvement, leading to further diagnostic evaluation. A skin biopsy from the cheek revealed a dense eosinophilic infiltrate in both the superficial and deep dermis, with evidence of folliculocentric inflammation (Figure 1d). Laboratory tests demonstrated marked eosinophilia (792 × 10^6^/L), but total immunoglobulin E was within the normal range. Viral serologies, including HIV, hepatitis B and hepatitis C, were negative.

**Figure 2:**
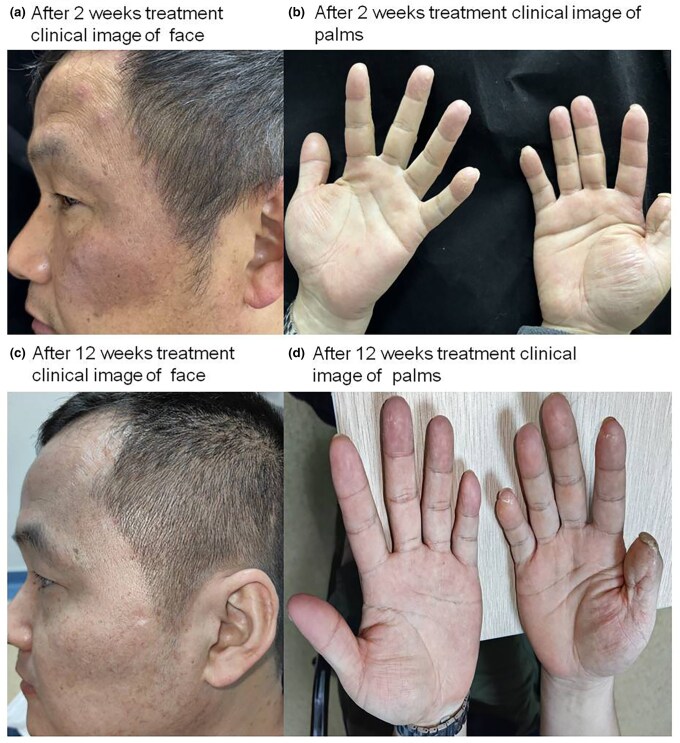
After 2 weeks treatment clinical image of (a) face and (b) palms, and after 12 weeks treatment clinical image of (c) face and (d) palms.

A diagnosis of eosinophilic pustular folliculitis (EPF) was made based on clinical, histopathological and laboratory findings. Given the patient’s refractory response to previous treatments, apremilast was initiated with a gradual titration over 5 days. Significant clinical improvement was noted within 2 weeks, with complete remission of skin lesions by week 12 (Figure 2), laboratory tests demonstrated that eosinophil count has decreased to the normal range (86 × 10^6^/L). The patient continued to be followed for 6 months without recurrence, and no adverse effects were reported during treatment.

## Discussion

EPF, particularly with palmoplantar involvement, presents a diagnostic challenge due to its rarity and resemblance to other dermatoses.^1^ In this case, the patient was initially misdiagnosed with palmoplantar pustulosis, a more common condition characterized by pustules on the palms and soles. However, the presence of eosinophilia and histopathological findings consistent with eosinophilic infiltration led to the correct diagnosis of EPF.

EPF has been historically treated with a wide range of therapies, including indomethacin, dapsone and topical or systemic corticosteroids.^5^ However, these treatments often provide only partial relief, and relapses are common. The underlying pathophysiology of EPF suggests that T helper 2 (Th2)-mediated eosinophilic inflammation plays a critical role in the disease process, making therapies that target Th2 cytokines, such as interleukin (IL)-4, IL-5 and IL-13, potentially effective.^6^ Immunohistochemistry or serology to these cytokines before and after treatment can be performed to verify the efficacy.

Emerging treatments for EPF include biologic agents and small-molecule inhibitors. Dupilumab, an IL-4 receptor antagonist, has been reported to successfully treat EPF by inhibiting Th2-driven inflammation.^6^ Similarly, mepolizumab (an anti-IL-5 monoclonal antibody) and adalimumab [a tumour necrosis factor alpha (TNF-α) inhibitor], have shown promise in reducing eosinophilic infiltration and alleviating symptoms of EPF.^7,8^ Abrocitinib, a Janus kinase inhibitor, has also been used successfully in recent cases of EPF.^9^

Apremilast, a PDE4 inhibitor, works by reducing the production of pro-inflammatory cytokines, including TNF-α, IL-17 and IL-23, which are involved in various inflammatory skin diseases.^10^ Its efficacy in this case is noteworthy, as the patient achieved complete remission without significant adverse effects. This adds to a growing body of evidence supporting the use of apremilast in eosinophilic dermatoses, particularly in cases refractory to conventional treatments.

This case highlights the importance of considering eosinophilic pustular folliculitis in the differential diagnosis of palmoplantar dermatoses. The successful treatment of this patient with apremilast underscores the potential of PDE4 inhibitors in managing EPF, a condition for which no standardized treatment guidelines currently exist. Further research is needed to explore the long-term efficacy and safety of apremilast and other emerging therapies in EPF.

## Data Availability

The data underlying this article are available upon request from the corresponding author.
